# A modified Delphi consensus statement on the role of biopsy in small renal masses

**DOI:** 10.1002/bco2.70018

**Published:** 2025-04-22

**Authors:** Darryl E. Bernstein, Hannah Warren, Joseph Santiapillai, Geraldine Fox, William H. Wildgoose, Grant D. Stewart, Jim Armitage, Pieter Le Roux, Frank X. Keeley, Nicholas Campain, Ben Challacombe, Hazel Warburton, Carlotta Palumbo, Riccardo Campi, Stjin H. J. Muselaers, Miles Walkden, Steve Bandula, Dominic Yu, Michael Gonsalves, My‐Anh Tran‐Dang, Nazanin Etessami, Pedro Oliveira, Anna Calio, Soha El‐Sheikh, Tze Wah, Axel Bex, Ravi Barod, Kurinchi Gurusamy, Maxine G. B. Tran

**Affiliations:** ^1^ Specialist Centre for Kidney Cancer Royal Free London NHS Foundation Trust London UK; ^2^ Division of Surgery and Interventional Science University College London London UK; ^3^ Kidney Cancer UK Surrey UK; ^4^ Department of Surgery University of Cambridge Cambridge UK; ^5^ Department of Urology Cambridge University Hospitals NHS Foundation Trust Cambridgeshire UK; ^6^ Epsom and St Hellier NHS Trust Surrey UK; ^7^ Bristol Urological Institute Bristol UK; ^8^ Royal Devon and Exeter NHS Foundation Trust Exeter UK; ^9^ Guy's and St Thomas' NHS Trust London UK; ^10^ Wythenshawe Hospital Manchester UK; ^11^ Università del Piemonte Orientale Norvara Italy; ^12^ Maggiore della Carità Hospital Novara Italy; ^13^ Unit of Urologic Robotic Minimally‐Invasive Surgery and Renal Transplantation of Careggi University Hospital Florence Italy; ^14^ Radboud University Medical Center Nijmegen The Netherlands; ^15^ University College London Hospital London UK; ^16^ Royal Free London NHS Foundation Trust London UK; ^17^ St. George's Hospital NHS Trust London UK; ^18^ Department of Pathology The Christie NHS Foundation Trust Manchester UK; ^19^ Department of Diagnostics and Public Health University of Verona Verona Italy; ^20^ Research Department of Pathology University College London UK; ^21^ The Leeds Teaching Hospital NHS Trust Leeds UK

**Keywords:** biopsy, consensus statement, kidney cancer, minimally invasive therapies, small renal mass

## Abstract

**Objective:**

To understand the variable utilisation of diagnostic biopsy for small renal masses (SRM) across the urology community, we worked with expert clinicians and patients to produce a consensus statement on the role of biopsy and to identify research gaps.

**Methods:**

In phase I, qualitative interviews were performed to identify potential statements on the role of biopsy and research gaps. In phase II, an expert panel including patients scored statements on a 9‐point scale through a modified Delphi process involving three rounds of web‐based surveys. Consensus was considered to have been reached when 70% of participants scored a statement greater than or equal to seven. Panel members could propose additional statements for consideration after the first round. Following the second round, a moderation meeting was held to discuss statements where threshold of agreement was not met.

**Results:**

In total, 35 participants were involved in this project and consisted of 23 clinicians and 12 patients, with 29 participants completing all three rounds. Overall, 18 statements reached consensus, 11 of which pertained to when and how a biopsy should be used in SRM management and 7 research recommendations to improve the evidence base for biopsy use.

**Conclusions and Clinical Implications:**

This Delphi consensus statement, co‐produced by patients and clinicians, provides best‐practice guidance on the current role of renal tumour biopsy, including offering biopsy prior to active treatment if the outcome would affect management and offering a second attempt should the first biopsy be non‐diagnostic. Priority areas for future research included studies to evaluate how a biopsy affects choice of treatment and patient anxiety.

## 1 INTRODUCTION

Since the 1970s, the finding of incidental renal tumours has increased due to widespread access and use of cross‐sectional imaging.^1^ More than 50% of diagnoses of kidney tumours are now at clinical stage T1a (also termed small renal mass [SRM]).^2^ However, despite earlier diagnosis, there has been little impact on overall kidney cancer mortality, suggesting an element of overdiagnosis and overtreatment.^1^ A nationwide nephrectomy audit carried out in the United Kingdom between 2013 and 2016 reported that partial nephrectomy carries a 20% peri‐operative complication risk and a 4% major complication risk,^3^ similar to international surgical series.^4^

Not all SRMs are cancer, and imaging alone cannot reliably distinguish between benign and cancerous tumours. Up to 30% of SRMs are benign, and the incidence can be as high as 36% in patients aged less than 40.^3^, ^5^ Currently, the only method of diagnosing a benign tumour before surgery is to have a renal tumour biopsy (RTB), which has a diagnostic rate of 89% and is a safe procedure with low complication rates of 2.5%.^6^ Studies also show RTB to be a cost‐effective and efficient use of healthcare resources.^7^ In the SRM setting, RTB results have been shown to influence patient preferences for treatment, favouring less invasive treatments for benign and indolent tumours.^8^

Current American Urological Association and European Association of Urology guidelines recommend a biopsy prior to commencing active surveillance, ablative therapy or systemic therapy, beyond which there is no guidance on the role of RTB in SRM management.^9^ However, despite favourable evidence, there is a low uptake of this diagnostic tool, with studies in England and the United States reporting similar rates of biopsy usage of 15%–20%.^10^, ^11^

This multidisciplinary project aimed to bring patients and clinicians together to produce a consensus statement on when to offer RTB and to identify areas of uncertainty and research gaps in its role in the management of SRM.

## 2 MATERIALS AND METHODS

### 2.1 The modified Delphi method

**FIGURE 1:**
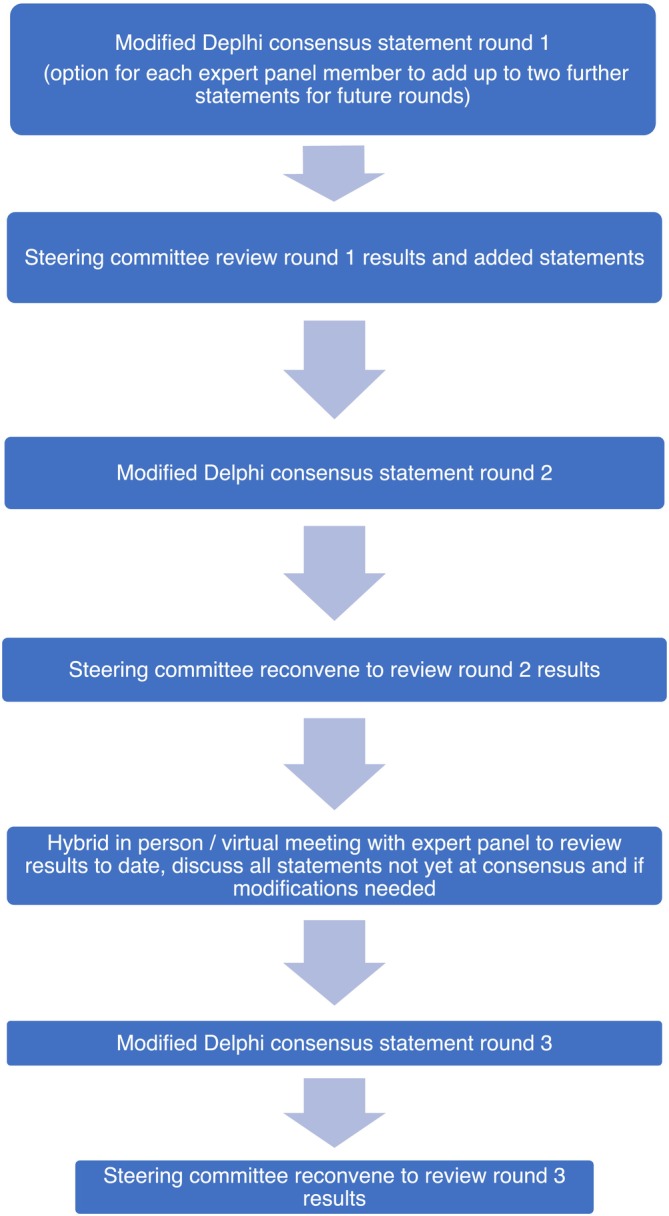
The modified Delphi process.

A modified Delphi consensus process was used for collating expert opinions for the study (Figure 1), according to recommended guidelines.^12^

### 2.2 Phase I

In Phase I, potential statements on the role of RTB were identified from qualitative semi‐structured interviews with patients, clinicians and allied health professionals in the study, ‘Identifying the facilitators and barriers to implementation of RTB in the diagnostic pathway for small renal masses’ (IFIT‐B).^13^ A project steering committee comprising two clinical experts in renal cancer, two patient representatives and a methodologist then convened to define the scope of the modified Delphi consensus statement and reviewed Phase I results for statement inclusion.

Twelve statements were selected to take forward to Phase II. Statements were divided into two categories: (1) when RTB should be offered and (2) evidence needed to support the uptake of RTB. Draft statements were reviewed for readability and the use of accessible language by patient representatives on the steering committee. A pragmatic decision was made to reduce the overall statement number to increase the likelihood of survey completion by panel members.

According to the HRA decision toolkit, this study was exempt from requiring REC review.

### 2.3 Phase II

#### 2.3.1 Expert panel selection

For Phase II, a purposive sample of opinion leaders in renal cell carcinoma (RCC) and patients was invited to form an expert panel. The panel (*N* = 52) comprised 38 international multidisciplinary experts (urologists, radiologists and pathologists) and 14 patients. Clinicians were identified through professional networks of the steering committee and the European Association of Urology Young Academic Urologists RCC working group. Patients were identified through the charity Kidney Cancer UK.

#### 2.3.2 Survey administration

Surveys were designed, collected and managed using the REDCap electronic data capture tools hosted at University College London.^14^, ^15^

Panel members were only invited to participate in the survey if they had completed the previous round. Reasons for survey dropout between rounds were collected.

#### 2.3.3 Delphi rounds

Surveys were modified in each of the three rounds of the process based on previous results.

In each of the three rounds, statements were scored from 1 to 9 to indicate agreement on a Likert scale (1 = *strongly disagree* to 9 = *strongly disagree*). Consensus for a statement in each round was considered to have been reached when ≥70% of participants scored a statement ≥7 to indicate agreement, 4–6 to indicate neutrality or ≤3 to indicate disagreement, respectively. If a statement reached consensus, it was not included in further rounds.

In round 1, expert panel members were invited to suggest up to two new statements for inclusion in subsequent rounds.

#### 2.3.4 In‐person/virtual hybrid meeting

An expert panel meeting was hosted between rounds two and three to review statements that had not reached consensus. Relevant literature to each statement was summarised and reviewed to determine whether the statement required modification.

## 3 RESULTS

### 3.1 Participation

Of 52 initial expert panel members invited, 35 participants completed round 1 (67% response rate), 32/35 completed round 2 (91% response rate) and 29/32 completed round 3 (83% response rate). The professional role of each participant completing the survey is presented in Table 1.

**TABLE 1:** Participation of expert panel across three rounds of modified Delphi consensus statement.

	Urologist	Pathologist	Radiologist	Patient	Total
Invited panel	46%	12%	15%	27%	
	24	6	8	14	52
	17 did not participate				
Round 1 panel	37.1%	14.3%	14.3%	34.3%	
	13	5	5	12	35
	3 participants dropped out between Round 1 and Round 2				
Round 2 panel	40.6%	15.6%	12.5%	31.3%	
	13	5	5	9	32
	4 participants dropped out between Round 2 and Round 3				
Round 3 panel	44.8%	17.2%	17.2%	20.7%	
	13	5	5	6	29

### 3.2 Statements that reached consensus

In total, 18/21 statements (86%) reached consensus (Table 2).

**TABLE 2:** Results of the 18 statements presented to the panel across three rounds of the modified Delphi consensus statement process that did reach consensus.

	Statement		Round 1	Round 2	Round 3
Statements reaching consensus in round 1	A biopsy should only be offered if it will help the clinician and patient decide on how the kidney tumour should be managed.	Scoring 7–9 Scoring 4–6 Scoring 1–3	77.1% 14.3% 8.6%		
	A patient should be able to access a biopsy service elsewhere if it is not offered at their local hospital.	Scoring 7–9 Scoring 4–6 Scoring 1–3	88.6% 2.9% 8.6%		
	A study is needed to show that having a biopsy changes the patient's choice of treatment (meaning, the patient may choose monitoring of the tumour if their biopsy shows that it is benign).	Scoring 7–9 Scoring 4–6 Scoring 1–3	80.0% 14.3% 5.7%		
	A study is needed to show that having a biopsy reduces the number of patients with benign tumours having surgery.	Scoring 7–9 Scoring 4–6 Scoring 1–3	82.9% 14.3% 2.8%		
	A study is needed to show that having a biopsy is an effective use of health resources.	Scoring 7–9 Scoring 4–6 Scoring 1–3	80.0% 17.1% 2.9%		
	A study is needed to show that having a biopsy improves patients' quality of life.	Scoring 7–9 Scoring 4–6 Scoring 1–3	74.3% 22.9% 2.8%		
Statements reaching consensus in round 2	Patients diagnosed with a solid kidney tumour on imaging should generally be offered a biopsy, if technically possible, to find out if the tumour is cancer before choosing treatment.	Scoring 7–9 Scoring 4–6 Scoring 1–3	68.8% 20.0% 11.4%	78.1% 9.4% 12.5%	
	A biopsy should be offered to patients whose tumours have grown or show changes on their scans.	Scoring 7–9 Scoring 4–6 Scoring 1–3	62.9% 31.4% 5.7%	84.4% 6.3% 9.4%	
	Evidence is needed which shows that classification of a tumour (grade and type) is the same at biopsy as when the tumour is removed surgically in its entirety (meaning the accuracy of the biopsy).	Scoring 7–9 Scoring 4–6 Scoring 1–3		71.8% 18.8% 9.4%	
	A study is needed which assesses the follow up of patients after a benign (non‐cancerous) biopsy result.	Scoring 7–9 Scoring 4–6 Scoring 1–3		71.8% 18.8% 9.4%	
	Further special tests, such as immunohistochemistry (staining for special proteins), should be considered when there is uncertainty in a biopsy result, to help provide more information.	Scoring 7–9 Scoring 4–6 Scoring 1–3		90.6% 3.1% 6.3%	
	A biopsy should be performed by an appropriately trained radiologist or urologist.	Scoring 7–9 Scoring 4–6 Scoring 1–3		93.7% 0.0% 6.3%	
Statements reaching consensus in round 3	A biopsy should NOT be offered to patients with cystic tumours (containing fluid) without a significant solid component, because there is a higher risk that the biopsy will not be successful.	Scoring 7–9 Scoring 4–6 Scoring 1–3	65.7% 22.9% 11.4%	68.8% 12.5% 18.8%	86.21% 0.00% 13.79%
	A second attempt at biopsy should be offered if the first attempt is inconclusive (non‐diagnostic).	Scoring 7–9 Scoring 4–6 Scoring 1–3	68.6% 20.0% 11.4%	65.6% 21.9% 12.5%	86.2% 6.9% 6.9%
	A biopsy should be considered prior to active treatment (surgery or ablation) for all small renal masses (less than 4 cm).	Scoring 7–9 Scoring 4–6 Scoring 1–3		59.4% 21.9% 18.8%	79.3% 6.9% 13.8%
	Before starting on active surveillance, biopsy should be considered to help tailor the follow up plan to the individual patient, e.g., frequency of imaging.	Scoring 7–9 Scoring 4–6 Scoring 1–3		59.4% 28.1% 12.5%	82.8% 10.3% 6.9%
	More research is needed to understand how having a renal biopsy affects patient anxiety.	Scoring 7–9 Scoring 4–6 Scoring 1–3		62.5% 28.1% 9.4%	79.3% 13.8% 6.9%
	More research is needed on the diagnostic rate of biopsy for different tumour sizes.	Scoring 7–9 Scoring 4–6 Scoring 1–3		59.4% 28.1% 12.5%	72.4% 20.7% 6.9%

### 3.3 Statements that did not reach consensus

In total, 3/21 statements (14%) did not reach consensus (Table 3).

**TABLE 3:** Results of the three statements presented to the panel across three rounds of the modified Delphi consensus statement process that did not reach consensus.

	Statement		Round 1	Round 2	Round 3
Statements not reaching consensus	A biopsy should not be offered to patients if there is high concern of tumour spread (seeding) from biopsy (for example there have been a few cases reported where cells from a particular type of tumour called a papillary tumour have spread into the path of the biopsy).	Scoring 7–9 Scoring 4–6 Scoring 1–3		34.4% 28.1% 37.5%	34.5% 20.7% 44.8%
	To encourage the use of kidney tumour biopsy, evidence that shows that the additional time to have the biopsy does not affect patient outcome is required (meaning, the delay caused by having a biopsy does not result in harm to the patient).	Scoring 7–9 Scoring 4–6 Scoring 1–3	68.6% 8.6% 22.9%	65.6% 12.5% 21.9%	58.6% 24.1% 17.2%
	To encourage the use of kidney tumour biopsy, there is a requirement for a study to examine the risk of tumour spread and subsequent cancer risk to the patient.	Scoring 7–9 Scoring 4–6 Scoring 1–3	65.7% 14.3% 20%	68.7% 18.8% 12.5%	58.6% 13.8% 27.6%

## 4 DISCUSSION

This is the first consensus statement of its kind, co‐developed by an international multi‐disciplinary team with patients as stakeholders, focusing on SRM RTB. The results of the modified Delphi process provide guidance on how RTB could be utilised in the SRM setting. It also identifies research gaps needed to improve RTB adoption.

### 4.1 Consensus statements

#### 4.1.1 When renal tumour biopsy should be offered

Statement 1: RTB should only be offered if it will help the clinician and patient decide on how the kidney tumour should be managed.
Statement 2: RTB should NOT be offered to patients with cystic tumours (containing fluid) without a significant solid component because there is a higher risk that RTB will not be successful.
Statement 3: A patient should be able to access RTB services elsewhere if it is not offered at their local hospital.

Statements reaching consensus reflect guideline recommendations on when RTB should be performed: prior to ablative therapy, systemic therapy and in certain patients starting active surveillance.^9^ Guidance additionally highlights that cystic renal masses should not be biopsied unless there are significant solid components, which this consensus statement agrees with.^9^ This consensus statement, however, extends guidance to scenarios not previously considered, starting with ensuring RTB is available regardless of geography.

Statement 4: Patients diagnosed with a solid kidney tumour on imaging should generally be offered RTB, if technically possible, to find out if the tumour is cancer before choosing treatment.
Statement 5: RTB should be considered prior to active treatment (surgery or ablation) for SRMs (less than 4 cm).
Statement 6: Before starting on active surveillance, RTB should be considered to help tailor the follow‐up plan to the individual patient, for example, frequency of imaging.

With the above statements reaching consensus, there is agreement that RTB should go further than current guidelines suggest, to personalise care and be a diagnostic tool available to all patients if technically feasible and not limited to specific populations. Statements reaching agreement support RTB being utilised to reduce unnecessary surgery, tailor patient‐specific active surveillance programmes, and assist the multi‐disciplinary team in deciding management strategies after patient diagnosis.^16^

Statement 7: A second attempt at RTB should be offered if the first attempt is inconclusive (non‐diagnostic).
Statement 8: RTB should be offered to patients whose tumours have grown or show changes on their scans.

Consensus was reached regarding repeat RTB, where it was felt that these should be considered in cases where tumours grow or change on active surveillance or if a first biopsy result is inconclusive. A systematic review of 20 studies showed that 80% of repeat biopsies are diagnostic.^17^

Overall, the statements highlight consensus areas where RTB could assist in effective and well‐informed surveillance and management plans, providing patients with management tailored to their condition, minimising overtreatment.

#### 4.1.2 Evidence needed to encourage biopsy use

Statement 9: A study is needed to show that having RTB changes the patient's choice of treatment (meaning, the patient may choose monitoring of the tumour if their biopsy shows that it is benign).
Statement 10: A study is needed to show that having RTB reduces the number of patients with benign tumours having surgery.
Statement 11: A study is needed to show that having RTB improves patients' quality of life.
Statement 12: More research is needed to understand how having RTB affects patient anxiety.

Research gaps highlighted in the Delphi were the need to review how biopsy affected patient anxiety and quality of life. As noted at the hybrid meeting, studies looking at the association of prostate biopsy and mental health exist, but there are limited studies for RTB.^18^ One single centre retrospective study has assessed the psychological impact of SRM management options, and though not directly assessing RTB, it found that those on surveillance with RTB proven malignancies have worse anxiety.^19^ Furthermore, studies have highlighted patient anxiety concerns associated with RTB missing cancer diagnoses or causing complications.^20^ Overall, consensus was that more research was needed into the mental health surrounding RTB to help inform future use.

Furthermore, consensus highlighted that more patient‐centred research was needed to show that RTB avoided intervention in cases of benign results and that patient treatment choices were altered (i.e., opting for surveillance when they would otherwise have opted for intervention). Though current publications highlight how biopsy reduces surgical intervention, more research is needed to show how benign findings specifically alter decision making.^11^, ^21^

Statement 13: A study is needed to show that having RTB is an effective use of health resources.
Statement 14: A study is needed that assesses the follow‐up of patients after a benign (non‐cancerous) RTB result.

From a performance perspective, there was consensus that economic data was required to show RTB was an effective use of healthcare resources. This would involve health economic analyses to show that RTB reduced the average cost to patient and healthcare systems over the duration of SRM management. Though models exist, real‐world data, based on contemporary SRM management protocols, would be beneficial, such as the single centre study which explored the value of advanced imaging such as (99m)Tc‐sestamibi scans.^22^ Previous larger analyses have been limited by considering only operative management of all malignant RTBs and excluding alternative ablative therapies.^7^, ^23^

The expert panel agreed there was a research gap in how benign RTBs should be followed up, highlighting the lack of uniform guidance.^9^

Statement 15: More research is needed on the diagnostic rate of RTB for different tumour sizes.
Statement 16: Evidence is needed that shows that classification of a tumour (grade and type) is the same at RTB as when the tumour is removed surgically in its entirety (meaning the accuracy of RTB).

With regard to biopsy results, consensus found that more research was needed to show the success of RTB performance prior to wider acceptance. This would be through studies assessing the diagnostic rate of RTB for various tumour sizes, with further studies showing that the pathology of RTB matched that of surgical tumour removal (grade/sub‐type). At the hybrid meeting, two small case series of less than 100 patients were discussed showing variable success of between 80% and 100% in SRM RTB depending on location, but more research was considered necessary to give clear guidance on how best to proceed based on anatomical location and size.^24^

#### 4.1.3 Other statements

Statement 17: RTB should be performed by an appropriately trained radiologist or urologist.

In the United Kingdom, RTB is primarily performed by radiologists, which may not be the case elsewhere. This statement, achieving consensus from clinicians and patients, acknowledges that it is also acceptable for urologists to undertake RTB, providing that they have been appropriately trained to do so.

Statement 18: Further special tests, such as immunohistochemistry (staining for special proteins), to help provide more information should be considered when there is uncertainty in the RTB result.

Though morphology alone is often sufficient for diagnosis after RTB, immunohistochemistry (IHC) is becoming increasingly useful when uncertainty exists, and an initial panel of CK7/AMACR/CAIX has been recommended for subtyping of the most common renal tumours.^25^ Moreover, innovative use of IHC, such as CD117, in conjunction with specific enhancement features on CT, can be useful in differentiating benign oncocytomas and indolent hybrid tumours from malignant chromophope RCC.^26^ Other adjuncts to RTB include nuclear medicine imaging, such as (99m)Tc‐sestamibi and girentuximab scans.^27^

From the patient's perspective, they felt all information possible should be gathered from RTB to avoid repeated procedures.

#### 4.1.4 Statements where consensus could not be reached

Statement 19: RTB should not be offered to patients if there is a high concern of tumour spread (seeding) from RTB. For example, there have been a few cases reported where cells from a particular type of tumour called a papillary tumour have spread into the path of the biopsy.

Consensus was not reached on whether biopsy should be offered in the setting of suspected papillary tumour RCC. This topic was discussed at length amongst the expert panel, where reference was made to a case series of six patients with papillary tumour seeding through their biopsy tract found on surgical resection specimens.^28^ The oncological sequelae of such seeding are unknown, with no local recurrence reported in patients in the aforementioned study (follow up ranged from 6 to 36 months) and discussion showed that this field has a significant research gap.^29^

Statement 20: To encourage the use of RTB, evidence that the additional time to have RTB does not affect patient outcomes is required (meaning, the delay caused by having RTB does not result in harm to the patient).

Studies discussed for this statement at the hybrid meeting noted the slow growth and metastasis rate of SRM, suggesting that enough natural history data already exists and that no further research was required.^30^

Statement 21: To encourage the use of RTB, there is a requirement for a study to examine the risk of tumour spread and subsequent cancer risk to the patient.

The expert panel felt that as seeding was such a rare event, the sample size requirements would be prohibitive for such a study.^17^

#### 4.1.5 Limitations

Notably, over one‐third of participants were urologists (Table 1), and one‐third of participants in rounds one and two were also patients. There was a drop in responses for round 3, and the steering committee noted that reasons provided for reduced participation included issues with ongoing illness amongst patients and time availability amongst clinicians.

Due to resource and logistic limitations, most of the expert panel was based in the United Kingdom, with only three from Europe, which could potentially limit applicability to other countries and health services.

## 5 CONCLUSIONS

In summary, we performed a modified Delphi consensus process to determine when RTB should be offered to patients with SRMs and the evidence still required to support its use. This collaborative project involved patients and multidisciplinary clinicians involved in the SRM patient management. The project identified 18 statements with an emphasis on broad themes: biopsy to aid personalisation of care.

## CONFLICT OF INTEREST STATEMENT

The authors declare there are no conflicts to disclose.
